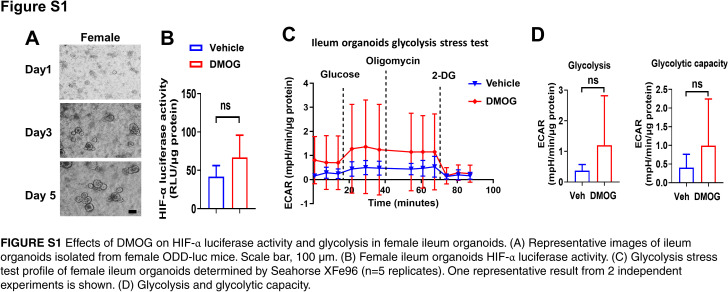# Intestine epithelial–specific hypoxia-inducible factor-1α overexpression ameliorates western diet–induced MASLD

**DOI:** 10.1097/HC9.0000000000000572

**Published:** 2024-11-25

**Authors:** Manman Xu, Madison S. Taylor, Bradford G. Hill, Xiaohong Li, Eric C. Rouchka, Craig J. McClain, Ming Song

**Affiliations:** 1 Department of Medicine, Division of Gastroenterology, Hepatology and Nutrition, University of Louisville School of Medicine, Louisville, Kentucky, USA; 2 Department of Medicine, Division of Environmental Medicine, Center for Cardiometabolic Science, Christina Lee Brown Envirome Institute, University of Louisville School of Medicine, Louisville, Kentucky, USA; 3 Kentucky Biomedical Research Infrastructure Network Bioinformatics Core, Louisville, Kentucky, USA; 4 Department of Biochemistry and Molecular Genetics, University of Louisville School of Medicine, Louisville, Kentucky, USA; 5 Department of Pharmacology and Toxicology, University of Louisville School of Medicine, Louisville, Kentucky, USA; 6 Hepatobiology & Toxicology Center, University of Louisville School of Medicine, Louisville, Kentucky, USA; 7 University of Louisville Alcohol Research Center, University of Louisville School of Medicine, Louisville, Kentucky, USA; 8 Robley Rex Veterans Affairs Medical Center, Louisville, Kentucky, USA

**Keywords:** DMOG, fructose, glucose tolerance, hepatic steatosis, RNA-seq

## Abstract

**Background:**

Intestine epithelial hypoxia-inducible factor-1α (HIF-1α) plays a critical role in maintaining gut barrier function. The aim of this study was to determine whether pharmacological or genetic activation of intestinal HIF-1α ameliorates western diet–induced metabolic dysfunction–associated steatotic liver disease.

**Methods:**

Metabolic effects of pharmacological activation of HIF-1α by dimethyloxalylglycine were evaluated in HIF-α luciferase reporter (ODD-luc) mice. Male and/or female intestinal epithelial–specific Hif1α overexpression mice (Hif1α^LSL/LSL;VilERcre^) and wild-type littermates (Hif1α^LSL/LSL^) were fed with regular chow diet, high fructose (HFr) or high-fat (60% Kcal) high-fructose diet (HFHFr) for 8 weeks. Metabolic phenotypes were profiled.

**Results:**

Dimethyloxalylglycine treatment led to increased intestine HIF-α luciferase activity and decreased blood glucose levels in HFr diet–fed male ODD-luc mice. Male Hif1α^LSL/LSL;VilERcre^ mice exhibited markedly improved glucose tolerance compared to Hif1α^LSL/LSL^ mice in response to HFr diet. Eight weeks HFHFr feeding led to obesity in both Hif1α^LSL/LSL;VilERcre^ and Hif1α^LSL/LSL^ mice. However, male Hif1α^LSL/LSL;VilERcre^ mice exhibited markedly attenuated hepatic steatosis along with reduced liver size and liver weight compared to male Hif1α^LSL/LSL^ mice. Moreover, HFHFr-induced systemic inflammatory responses were mitigated in male Hif1α^LSL/LSL;VilERcre^ mice compared to male Hif1α^LSL/LSL^ mice, and those responses were not evident in female mice. Ileum RNA-seq analysis revealed that glycolysis/gluconeogenesis was up in male Hif1α^LSL/LSL;VilERcre^ mice, accompanied by increased epithelial cell proliferation. Moreover, an in vitro study showed that HIF stabilization enhances glycolysis in intestine organoids.

**Conclusions:**

Our data provide evidence that pharmacological or genetic activation of intestinal HIF-1α markedly ameliorates western diet–induced metabolic dysfunction–associated steatotic liver disease in a sex-dependent manner. The underlying mechanism is likely attributed to HIF-1α activation–induced upregulation of glycolysis, which, in turn, leads to enhanced epithelial cell proliferation and augmented gut barrier function.

## INTRODUCTION

Metabolic dysfunction–associated steatotic liver disease (MASLD) is the most common liver disease and affects more than 30% of the population in the United States and worldwide.^1^ Crosstalk between the gut microbiota and liver controls gastrointestinal and liver health.^2^ Emerging evidence suggests that impaired gut barrier function plays a critical role in the development of MASLD.^3^^–^^5^ Specific nutrients, including fat and fructose, have been shown to disrupt gut barrier function and facilitate the progression of obesity and MASLD through alterations of gut microbial activities.^6^^,^^7^ The impaired gut barrier integrity leads to bacteria translocation and systemic inflammation,^8^ which play a causal role in the development of metabolic syndrome and MASLD.^9^

The gut barrier is composed of several layers, including the mucus layer, the epithelial cell layer, and the vascular barrier.^4^ Stimulation of epithelial cell proliferation by activation of signaling pathways that control tissue regeneration has been shown to rescue gut barrier function in high-fructose diet–fed mice and, in turn, inhibit hepatic de novo lipogenesis and subsequent hepatic steatosis.^7^^,^^10^

HIF is a master transcriptional factor in the adaption to hypoxia and plays a critical role in the maintenance of gut barrier function.^11^ Physiological hypoxia is an important characteristic of a healthy gut and is maintained, in part, by the crosstalk between the host and the gut microbiota. The gut microbiota–generated short-chain fatty acid, butyrate, is an important energy source of colonocytes. Butyrate is metabolized through mitochondrial β-oxidation and consumes oxygen, thereby depleting oxygen, which consequently activates HIF-1 and its target genes. This, in turn, favors normal barrier function.^11^ Conversely, depletion of gut microbiota or butyrate-producing bacteria leads to metabolic reprogramming from β-oxidation to anaerobic glycolysis and consumes less oxygen, which disrupts intestine physiological hypoxia and results in pathogen colonization and impaired barrier function.^11^^,^^12^

HIF-1 activation protects against experimental colitis with improved gut epithelial barrier function,^13^ yet its role in MASLD remains elusive. The aim of this study is to test whether intestine HIF-1 activation is beneficial in the improvement of MASLD induced by fructose-containing western diet. Here, we report that pharmacological activation of HIF-1 and genetic overexpression of intestine epithelial HIF-1 markedly improves western diet–induced MASLD in murine models, likely through upregulation of glycolysis, leading to intestine epithelial cell proliferation and enhanced gut barrier function.

## METHODS

A detailed description of the methodology is provided in the Supplemental Methods, http://links.lww.com/HC9/B80.

### Statistical analysis

Data are expressed as mean ± SD and were analyzed using unpaired *t* test, Mann-Whitney test, 1-way or 2-way ANOVA followed by the Tukey multiple comparison test. Differences at *p* ≤ 0.05 were considered to be statistically significant.

## RESULTS

### Pharmacological activation of hypoxia-inducible factor-1α activity abrogated dietary high-fructose–induced hyperglycemia

**FIGURE 1:**
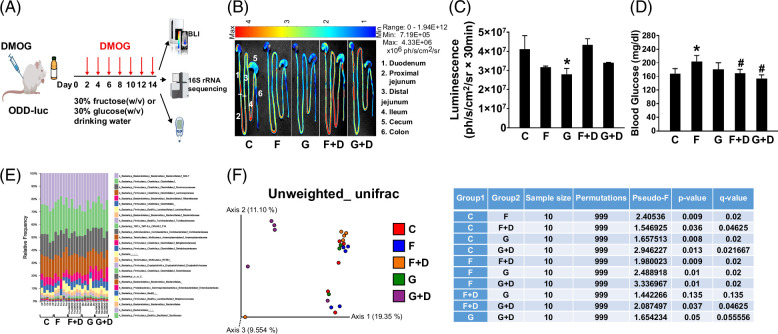
Pharmacological activation of intestine HIF-1α improves blood glucose associated with altered gut microbiome in male ODD-luc mice. (A) Experimental design for pharmacological activation of intestine HIF-1α on the alteration of metabolic phenotypes (created with BioRender.com). (B) Representative pictures of BLI at 15 minutes after D-luciferin injection. (C) Quantification of BLI. (D) Blood glucose (nonfasted). (E) Cecum gut microbiota composition at the family level (16S rRNA sequencing results), and (F) β-diversity (unweighted-unifrac). Data represent means ± SD (n = 5), *, ^#^ *p* < 0.05, 1-way ANOVA. * versus C, # versus F. Abbreviations: BLI, bioluminescence imaging; C, control; D, DMOG; F, fructose; G, glucose; HIF-1α, hypoxia-inducible factor-1α.

To test whether pharmacological activation of HIF-1 improves the dietary fructose–induced metabolic phenotype and gut microbiota dysbiosis, we treated male ODD-luc mice with the hydroxylase inhibitor, dimethyloxalylglycine (DMOG), through i.p. injection, a protocol that has been shown to cause HIF-1 activation in vivo.^13^ Meanwhile, the mice were ad libitum fed with 30% fructose or 30% glucose (w/v) in drinking water for 2 weeks (Figure 1A). DMOG-induced intestinal hypoxia-inducible factor-1α (HIF-1α) activation was validated by HIF-1α luciferase activity (Figures 1B, C). Notably, DMOG treatment abrogated high fructose (HFr)-induced hyperglycemia (Figure 1D). Moreover, DMOG treatment led to a remarkable alteration of gut microbiota composition in fructose-fed mice (*p* = 0.009) and a trend of alteration in glucose-fed mice (*p* = 0.05) as shown by β-diversity (unweighted_unifrac) and taxonomic composition (Figures 1E, F). These data suggest that systemic DMOG treatment leads to intestinal HIF-1 activation, which is associated with an improved metabolic phenotype. Therefore, the data provided evidence of therapeutic potential of DMOG in the treatment of fructose-induced metabolic disorders.

### Intestine-specific HIF-1α overexpression improves HFr-induced glucose intolerance

**FIGURE 2:**
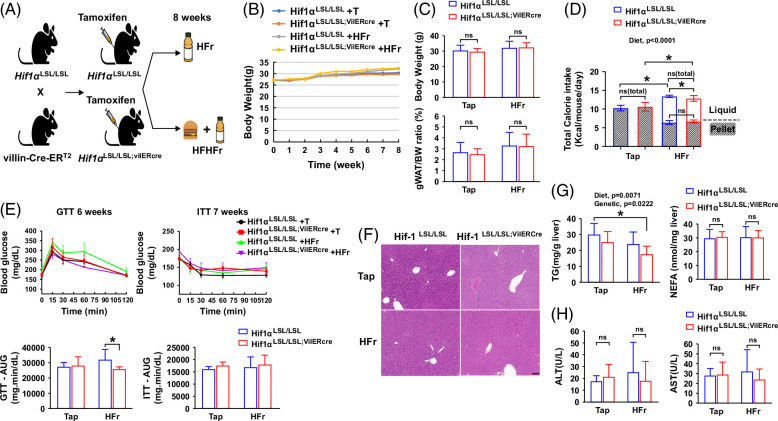
Intestine-specific HIF-1α overexpression improves HFr diet–induced glucose intolerance in male mice. Male Hif1α^LSL/LSL^ and Hif1α^LSL/LSL;VilERcre^ mice were fed with HFr for 8 weeks. (A) Schematic overview of generation of intestinal epithelial–specific HIF-1α overexpression mice and diet-induced MASLD models (created with BioRender.com). (B) BW growth trajectory. (C) BW and gWAT/BW ratio. (D) Calorie intake (n = 8–9, male). (E) GTT, ITT, and calculated AUC (n = 4–5). (F) Representative photomicrographs of the H&E staining of the liver section. Scale bar, 100 μm. (G) Hepatic TG and NEFA (n = 8–9). (H) Plasma ALT and AST (n = 8–9). Data represent means ± SD. Statistical significance was set to *p < 0.05, 2-way ANOVA followed with Tukey’s multiple comparison test or Mann-Whitney test. Abbreviations: BW, body weight; GTT, glucose tolerance test; gWAT, gonadal white adipose tissue; H&E, hematoxylin and eosin; HFr, high-fructose; HIF-1α, hypoxia-inducible factor-1α; ITT, insulin tolerance test; NEFA, nonesterified fatty acid; TG, triglyceride.

To validate the role of intestinal HIF-1 in the regulation of fructose-induced MASLD and metabolic phenotypes, male intestine–specific HIF-1α overexpression mice were chronically fed with a high-fructose diet (HFr) for 8 weeks (Figure 2A). Eight-week HFr feeding did not lead to obvious obesity in both Hif1α^LSL/LSL^ and Hif1α^LSL/LSL;VilERcre^ male mice (Figures 2B, C) despite increased calorie intake in Hif1α^LSL/LSL;VilERcre^ mice (Figure 2D). To determine the effect of intestine-specific HIF-1α overexpression on glucose metabolism, we performed a glucose tolerance test and insulin tolerance test on mice exposed to the experimental diet for 5 and 7 weeks, respectively. Glucose tolerance was significantly improved in Hif1α^LSL/LSL;VilERcre^ mice exposed to HFr compared to the control (Hif1α^LSL/LSL^) mice, while insulin action was not altered by intestine-specific HIF-1α overexpression as shown by glucose tolerance test and insulin tolerance test (Figure 2E). Eight-week HFr feeding did not induce obvious hepatic steatosis (Figures 2F, G) and liver injury as shown by plasma ALT and AST (Figure 2H), in both Hif1α^LSL/LSL^ and Hif1α^LSL/LSL;VilERcre^ mice. Nonetheless, hepatic triglyceride content was significantly lower in the Hif1α^LSL/LSL;VilERcre^ mice exposed to HFr compared to the control mice (Figure 2G). Overall, these data suggest that intestine-specific HIF-1α overexpression is protective against HFr-induced metabolic phenotypes.

### Intestine-specific HIF-1α overexpression attenuates high-fat high-fructose-induced steatosis in male mice

**FIGURE 3:**
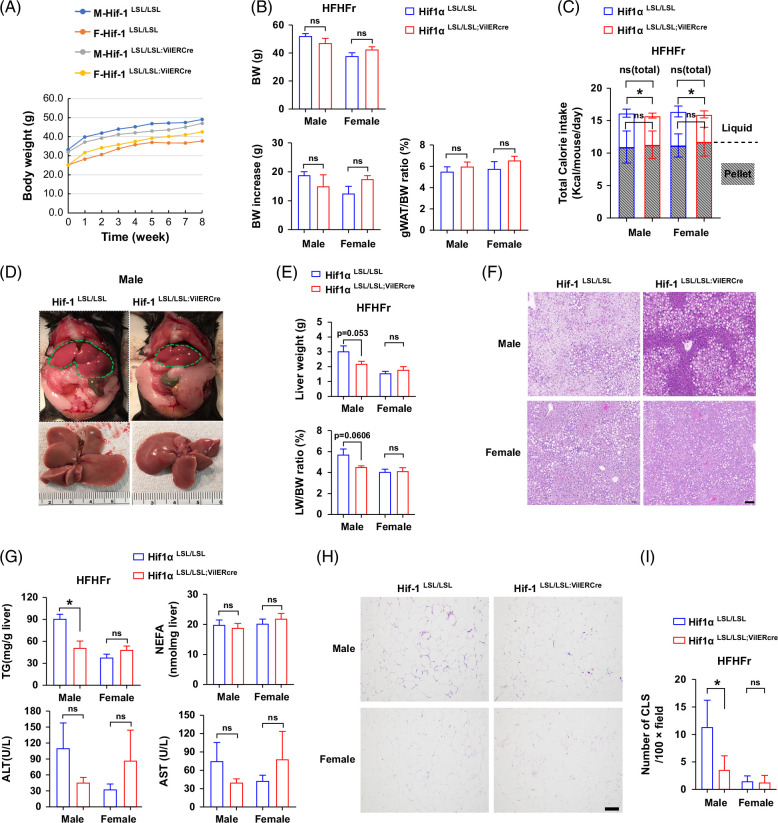
Intestine-specific HIF-1α overexpression attenuates HFHFr-induced steatosis in male mice. Male and female Hif1α^LSL/LSL^ and Hif1α^LSL/LSL;VilERcre^ mice were fed with HFHFr for 8 weeks. (A) BW growth trajectory. (B) BW, BW increase, and gWAT/BW ratio (n = 6–7, male; n = 5, female). (C) Calorie intake (n = 6–7, male; n = 5, female). (D) Representative gross morphology of liver in male Hif1α^LSL/LSL^ and Hif1α^LSL/LSL;VilERcre^ mice. The green dotted line defines the liver. (E) LW and LW/BW ratio (n = 6–7, male; n = 5, female). (F) Representative photomicrographs of H&E staining of liver section. Scale bar, 100 μm. (G) Liver TG, NEFA, plasma ALT, and AST (n = 6–7, male; n = 5, female). (H) Representative photomicrographs of the H&E staining of gWAT section. Scale bar, 100 μm. (I) Quantitation of CLSs in gWAT (5–10 field/mouse, n = 3). Data represent means ± SD. Statistical significance was set to *p < 0.05, Unpaired *t* test. Abbreviations: BW, body weight; CLS, crown-like structure; gWAT, gonadal white adipose tissue; H&E, hematoxylin and eosin; HFHFr, high-fat high-fructose; HIFα, hypoxia-inducible factor-1α; LW, liver weight; NEFA, nonesterified fatty acid; TG, triglyceride.

To further validate the beneficial role of intestine-specific HIF-1α overexpression on the improvement of metabolic phenotypes, we fed mice with HFr in the presence of a high-fat diet (60% Kcal fat) (HFHFr), which is known to induce obesity and MASLD.^14^ Eight-week HFHFr feeding led to obvious obesity in both Hif1α^LSL/LSL^ and Hif1α^LSL/LSL;VilERcre^ male mice (body weight [BW], 52.3 vs. 30.3 g, HFHFr-fed versus chow-fed male Hif1α^LSL/LSL^ mice). However, intestine-specific HIF-1α overexpression did not lead to significant differences in BW, BW increase, and gonadal white adipose tissue/BW ratio in both male and female mice (Figures 3A, B). There were no significant differences in the total calorie intake between Hif1α^LSL/LSL^ and Hif1α^LSL/LSL;VilERcre^ mice in both males and females, despite a mild but significant increased calorie intake in terms of fructose in Hif1α^LSL/LSL^ mice (Figure 3C). Liver size and liver weight trended lower in the male Hif1α^LSL/LSL;VilERcre^ mice compared to the male Hif1α^LSL/LSL^ mice. Female mice exhibited a lower liver weight and liver weight/BW ratio compared to the male mice, and no significant differences were observed between female Hif1α^LSL/LSL^ and Hif1α^LSL/LSL;VilERcre^ mice (Figures 3D, E). Eight-week HFHFr feeding led to severe hepatic steatosis in male Hif1α^LSL/LSL^ mice, and that was attenuated in Hif1α^LSL/LSL;VilERcre^ mice. Female mice developed moderate steatosis when exposed to the HFHFr diet. No significant differences were observed in hepatic steatosis between female Hif1α^LSL/LSL^ and Hif1α^LSL/LSL;VilERcre^ mice. Plasma ALT and AST trended to decrease in male Hif1α^LSL/LSL;VilERcre^ mice. Female mice displayed opposite trends in terms of ALT and AST (Figures 3F, G). However, no statistical difference was reached. Of note, HFHFr feeding led to obvious adipose tissue inflammation in male Hif1α^LSL/LSL^ mice as shown by pronounced crown-like structures and that was markedly reduced in male Hif1α^LSL/LSL;VilERcre^ mice. No obvious adipose tissue inflammation was observed in either Hif1α^LSL/LSL^ or Hif1α^LSL/LSL;VilERcre^ female mice (Figures 3H, I). Collectively, these data further demonstrated the beneficial role of intestine-specific HIF-1 overexpression in protecting against western diet–induced MASLD.

### Intestine-specific HIF-1α overexpression attenuates HFHFr-induced systemic inflammatory response

**FIGURE 4:**
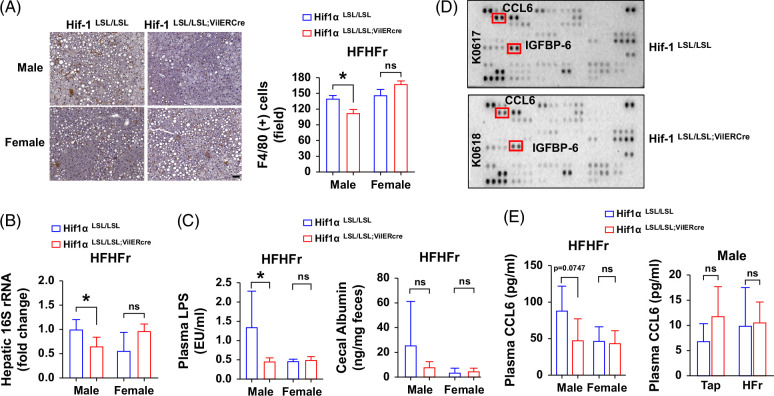
Intestine-specific HIF-1α overexpression attenuates HFHFr-induced systemic inflammatory response and bacteria translocation in male mice. Male and female Hif1α^LSL/LSL^ and Hif1α^LSL/LSL;VilERcre^ mice were fed with HFHFr for 8 weeks. (A) Representative photomicrographs (left panel) and quantitation (right panel) of IHC staining of F4/80 in the liver section (n = 6, male; n = 4–5, female). Scale bar, 50 μm. (B) Hepatic total 16S bacterial rRNAs normalized to host 18S rRNA (n = 5–6, male or female). (C) Plasma LPS and cecal albumin (n = 4–7, male or female); (D) Representative photomicrographs of cytokine array of plasma in male mice (n = 6/group, 1 dot represents the pulled results of 3 different plasma samples from the same group). (E) Plasma CCL6 level by ELISA (n = 4–6, male; n = 5, female). Left, HFHFr; Right, HFr. Data represent means ± SD. Statistical significance was set to *p < 0.05, Unpaired *t* test. Abbreviations: CCL6, C-C motif chemokine ligand 6; HFHFr, high-fat high-fructose; HIFα, hypoxia-inducible factor-1α; IGFBP6, insulin-like growth factor binding protein 6; IHC, immunohistochemical; LPS, lipopolysaccharide.

A major function of HIF-1 in the intestine is to maintain gut barrier function and integrity.^11^ Hence, we proposed that forced overexpression of intestine HIF-1α would enhance the gut barrier function. To this end, we sought to examine hepatic and systemic inflammatory responses and bacteria translocation. Our data show that the number of hepatic macrophages (F4/80 positive staining cell) was markedly reduced in male Hif1α^LSL/LSL;VilERcre^ mice compared to Hif1α^LSL/LSL^ mice when chronically fed with HFHFr. However, the difference was not obvious in female mice (Figure 4A). In line with this, real time quantitative PCR results showed that hepatic bacterial DNA copy number was significantly reduced in male Hif1α^LSL/LSL;VilERcre^ mice compared to the male Hif1α^LSL/LSL^ mice, suggesting reduced bacteria translocation and that effect was not evident in female mice (Figure 4B). Consistent with this, plasma lipopolysaccharide was significantly decreased in male Hif1α^LSL/LSL;VilERcre^ mice compared to the male Hif1α^LSL/LSL^ mice, and no differences were observed in female mice. Cecal albumin content, which is a reflection of intestinal permeability,^15^ was higher in male Hif1α^LSL/LSL^ mice than in male Hif1α^LSL/LSL;VilERcre^ mice, although the difference did not reach statistical significance. There was no obvious difference in female mice in terms of cecal albumin content (Figure 4C). Using a cytokine array, we identified that plasma chemokines, C-C motif chemokine ligand 6 (CCL6) and insulin-like growth factor binding protein 6, were markedly decreased in male Hif1α^LSL/LSL;VilERcre^ mice compared to the male Hif1α^LSL/LSL^ mice, and the changes of plasma CCL6 level were validated by ELISA. However, plasma CCL6 was detected at a very low level in chow-fed mice, and no significant differences were observed between Hif1α^LSL/LSL^ and Hif1α^LSL/LSL;VilERcre^ male mice in response to HFr feeding (Figures 4D, E). Collectively, these data suggest that intestine HIF-1α overexpression results in augmented gut barrier function, as shown by decreased hepatic and systemic inflammatory response and bacterial translocation when exposed to HFHFr.

### Intestine-specific HIF-1α overexpression leads to metabolic reprogramming toward glycolysis

**FIGURE 5:**
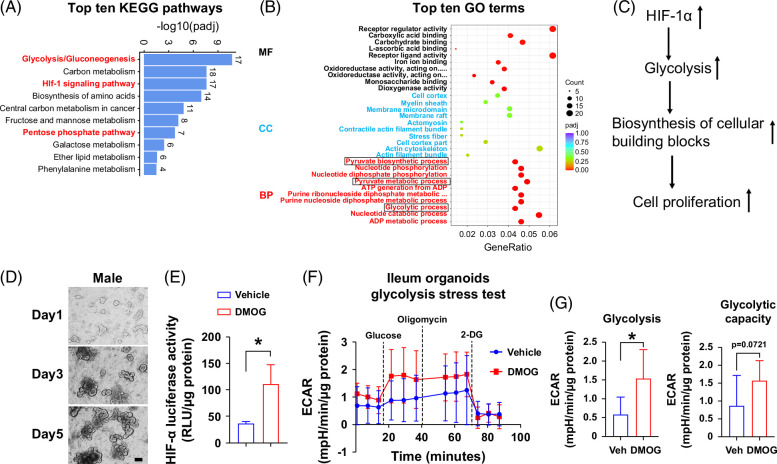
Intestinespecific HIF-1α overexpression leads to metabolic reprogramming toward glycolysis. Ileum RNA-seq analysis revealed metabolic reprogramming toward glycolysis by intestine-specific HIF-1 overexpression in male mice. (A) Top 10 KEGG pathways. (B) Top 10 GO terms (n = 3/group, male). (C) Schematic of working hypothesis. (D) Representative images of ileum organoids isolated from male ODD-luc mice. Scale bar, 100 μm. (E) Male ileum organoids HIF-α luciferase activity treated with 1 mM DMOG for 24 hours. (F) Glycolysis stress test profile of ileum organoids determined by Seahorse XFe96 (n = 5 replicates). One representative result from 2 independent experiments is shown. (G) Glycolysis and glycolytic capacity. Statistical significance was set to *p<0.05, Unpaired t test. Abbreviations: DMOG, dimethyloxalylglycine; GO, gene ontology; HIFα, hypoxia-inducible factor-1α; KEGG, Kyoto Encyclopedia of Genes and Genomes.

To understand the mechanisms of intestine HIF-1α overexpression on gut barrier function, we performed ileum bulk RNA-seq analysis in male Hif1α^LSL/LSL^ and Hif1α^LSL/LSL;VilERcre^ mice. Gene ontology and Kyoto Encyclopedia of Genes and Genomes (KEGG) pathway enrichment analyses were performed to identify the signaling pathways altered by intestine HIF-1α overexpression. Among the top 10 upregulated KEGG pathways by intestine HIF-1α overexpression, glycolysis was ranked number one. In addition, the pentose phosphate pathway was also upregulated. Intestine HIF-1α overexpression was validated by the upregulated HIF-1 signaling pathway in KEGG enrichment analysis (Figure 5A). In line with this, the glycolytic process and pyruvate metabolic process were also in the top gene ontology terms (Figure 5B). Given that an important biological function of glycolysis is to increase the synthesis of cellular building blocks and promote cell proliferation,^16^ this leads to the hypothesis that intestine HIF-1α overexpression rewires metabolic reprogramming toward glycolysis, which, in turn, promotes epithelial cell proliferation and subsequently enhances gut barrier function (Figure 5C). To test this hypothesis, we performed a Seahorse glycolysis stress test using isolated ileum organoids from male and female HIF-α reporter mice (Figure 5D). Pretreatment with DMOG led to significantly enhanced HIF-α luciferase activity and glycolysis in male ileum organoids (Figure 5E–G). However, these effects were not significant in female ileum organoids (Supplemental Figure S1, http://links.lww.com/HC9/B81), likely owing to the loss of estrogen effect in the vitro system.^17^

### Intestine-specific HIF-1α overexpression promotes epithelial cell proliferation and improves intestinal morphology

**FIGURE 6:**
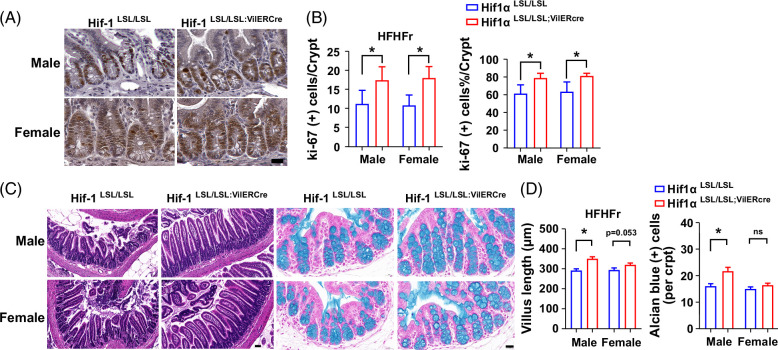
Intestinespecific HIF-1α overexpression promotes epithelial cell proliferation and improves intestinal morphology. (A) Representative photomicrographs of IHC staining of Ki-67 in the ileum section. Scale bar, 20 μm. (B) Quantitation of Ki-67–positive cell numbers and percentages in ileum crypts (30–50 crypts/10–15 field/mouse, n = 3). (C) Representative photomicrographs of H&E staining of the ileum (left panel, scale bar 50 μm) and Alcian blue staining of colon section (right panel, scale bar 20 μm) (n = 5–7, male; n = 5, female). (D) Villus length of ileum and goblet cell number quantification. Data represent means ± SD. Statistical significance was set to *p < 0.05, Unpaired *t* test. Abbreviations: H&E, hematoxylin and eosin; HIFα, hypoxia-inducible factor-1α; IHC, immunohistochemical

To validate that intestine epithelial HIF-1α overexpression promotes epithelial cell proliferation, we performed Ki-67 immunohistochemical staining. The number and percentage of Ki-67–positive staining cells in the crypts of ileum were significantly increased by intestine epithelial HIF-1α overexpression in both males and females (Figures 6A, B). Consistent with this, the villus length of the ileum was markedly increased in the male Hif1α^LSL/LSL;VilERcre^ mice accompanied with a more tidy alignment along the intestinal lumen compared to the male Hif1α^LSL/LSL^ mice, as shown by H&E staining. Similar findings were present in female mice. Moreover, there were more abundant goblet cells in the colons of male Hif1α^LSL/LSL;VilERcre^ mice compared to Hif1α^LSL/LSL^ male mice, as shown by Alcian blue positive staining cells. Although the number of goblet cells was not significantly changed in female mice, the size of mucin-secreting granules was larger in Hif1α^LSL/LSL;VilERcre^ mice than in Hif1α^LSL/LSL^ mice (Figures 6C, D). Collectively, intestine epithelial HIF-1α overexpression led to a significantly increased epithelial cell proliferation and markedly improvement of intestinal morphology, implying enhanced gut barrier function.

### The effect of sex on intestinal HIF activity in response to dietary fructose

**FIGURE 7:**
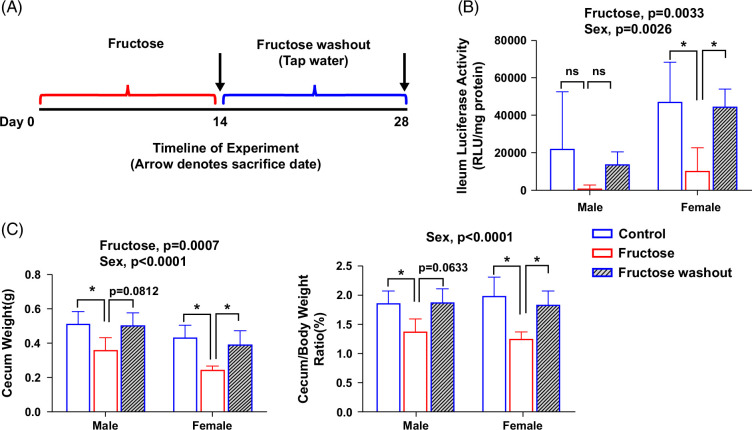
The effect of sex on intestinal HIF activity in response to dietary fructose. (A) Schematic timeline of the experiment. (B) Ileum HIF-α luciferase activity. (C) Cecum weight. (D) Cecum/body weight ratio. Data represent means ± SD (n = 5 except n = 3 in male fructose washout). *p < 0.05, 2-way ANOVA followed with the Tukey multiple comparison test. Abbreviation: HIFα, hypoxia-inducible factor-1α.

To examine the effect of sex on tissue-specific HIF activity, we measured the intestine HIF-α luciferase activity in both male and female ODD-luc mice in response to fructose feeding and fructose washout (Figure 7A). We found that basal HIF-α luciferase activity of ileum epithelium is higher in female mice than in male mice. Fructose feeding led to reduced HIF-α luciferase activity and that was restored with fructose washout. The changes in HIF-α luciferase activity were more pronounced in female mice (Figure 7B). Moreover, the alterations of ileum luciferase activity parallel the alterations of cecum weight and cecum/BW ratio (Figure 7C), which is an indicator of gut bacteria mass,^18^ suggesting a link between HIF-1 activity and gut microbiota.

## DISCUSSION

Accumulated evidence has shown that gut barrier dysfunction plays a causal role in the development of MASLD as well as in ALD through the gut-liver axis. A variety of strategies aimed at the improvement of gut barrier function have been shown to be promising in the attenuation of metabolic diseases. HIF-1 is known to regulate gut barrier function through a cascade of gene transcription that is directly involved in gut barrier protective function, including *TFF3*, *CLDN1*, *ABCB1*, *MUC-3*, *LL-37*, and *β-defensin*.^19^ Intestine epithelial Hif1α deficiency exacerbates alcohol-associated liver disease by worsening gut barrier function.^20^ Conversely, pharmacological activation of HIF-1 improves gut barrier function and protects against murine colitis.^13^ In the current study, we demonstrated that pharmacological activation of HIF-1 and genetic forced intestine HIF-1 overexpression protects against western diet–induced MASLD and was associated with reduced systemic inflammatory response and hepatic bacterial translocation. Our study suggests that intestine HIF-1 activation is a promising strategy for the treatment of MASLD. Of note, while both prolyl hydroxylase (PHD) and von Hipple Lindau inhibition result in HIF activation, PHD inhibitors or genetic disruption of PHDs have been shown to effectively activate HIF-1α and to improve gut barrier function in murine colitis models.^13^^,^^21^^–^^23^ However, disruption of von Hipple Lindau in the gut epithelium leads to stabilization of both HIF-1α and HIF-2α, with the proinflammatory effects of HIF-2α being dominant in the colitis model.^23^ In the current study, we demonstrated the beneficial effects of PHD inhibitors in the HFr-induced metabolic disorder model.

Under physiological hypoxia of the intestine lumen, HIF is stabilized through oxygen depletion owing to mitochondrial β-oxidation of butyrate, a metabolic process which consumes oxygen at a higher rate.^11^ Depletion of butyrate-producing bacteria with broad-spectrum or specific antibiotics reprograms metabolism toward glycolysis, diminishes physiologic hypoxia, and leads to reduced intestinal HIF stabilization and impaired gut barrier function that can be rescued by butyrate supplementation.^11^^,^^24^^,^^25^ These studies highlight the importance of metabolic regulation of intestine HIF activity under physiological conditions. However, our RNA-seq data show that forced intestine HIF-1 overexpression resulted in enhanced glycolysis in the ileum of transgenic mice. This is not surprising since it is well-known that a major role of HIF-1 is the regulation of glycolysis.^26^ Unlike in physiological conditions, this metabolic reprogramming led to a marked improvement in intestine morphology and reduced bacterial translocation and inflammatory responses, suggesting enhanced gut barrier function.

The biological significance of glycolysis in the regulation of proliferation is well documented.^16^ This leads to the hypothesis that upregulated glycolysis promotes cell proliferation, in turn enhancing gut barrier function. This is supported by increased Ki-67–positive staining cells and improved intestine morphology as a result of intestine HIF-1α overexpression (Figure 6). In line with this, stimulation of epithelial cell proliferation by targeting gp130 signaling confers protection of gut barrier function and improves fructose-induced steatosis.^7^^,^^10^ While most studies focus on the role of HIF-1 on the transcriptional regulation of tight junction, antimicrobial peptide, and mucosa protective genes, our study provides evidence that intestine HIF-1 overexpression–induced metabolic reprogramming toward glycolysis may lead to intestine epithelial cell proliferation and enhanced gut barrier function. Nonetheless, more detailed studies are needed to validate this concept.

While HIF-1α is ubiquitously expressed across normal tissues and cell types,^27^^,^^28^ its role appears to be cell type–specific and context-specific. Contrary to the role of HIF-1 in the intestine epithelial cells, hepatocyte HIF-1 deficiency protects against high-fat diet–induced MASLD.^29^ However, Ochiai et al^30^ reported that hepatocyte HIF-1 deficiency exacerbates insulin resistance and glucose intolerance in mice fed with a high-fat high-sucrose diet. Moreover, the debate also exists regarding the role of hepatocyte HIF-1 in alcohol-induced hepatic steatosis and liver injury,^31^^,^^32^ highlighting the complexity of HIF signaling in the context of metabolic liver diseases.

Similarly, the role of HIF-1 in the intestine is cell-type and disease-dependent. A previous study showed that endothelial-specific deletion of HIF-1α protects against radiation-induced enteritis, whereas epithelial-specific deletion of HIF-1α does not.^33^ Given that the primary lesion initiated by intestinal radiation damage is endothelial cell apoptosis, it is likely that HIF-1α plays a critical in the regulation of this process. In addition to epithelial and endothelial cells, the gut microenvironment is composed of a variety of cell types, including innate immune cells and adaptive immune cells. The communication between these cells is essential to maintain intestinal homeostasis and gut barrier function.^34^ Accumulated evidence suggests that HIF plays a central role in the maintenance of gut barrier function by integration of a variety of cell types.^35^ For instance, HIF-1α–dependent induction of FoxP3 enhanced anti-inflammatory effect of regulatory T cells and protected against T-cell–mediated colitis.^36^ Moreover, HIF-1 signaling in dendritic cells indirectly contributes to the activation of regulatory T cells.^37^ On the other hand, HIF-1–dependent IL-22 production in group 3 innate lymphoid cells contributes to the protection of *C. rodentium*–induced colitis.^38^ Deletion of HIF-1 in myeloid cells leads to unresolved inflammation in a DSS-induced colitis murine model.^39^ However, HIF-1 deficiency suppresses inflammation in murine models of arthritis and skin inflammation owing to metabolic reprogramming toward glycolysis.^40^ Given the cell-type–specific role of HIF-1 in gut barrier function, it will be interesting to examine the effects of gain- and loss of HIF-1α in the different cell types of intestine in the context of MASLD.

Sex difference in patients with MASLD has been noted, with women of reproductive age being protected from MASLD and having a 50% lower risk compared to men.^41^ In our study, the sex difference in HFHFr-induced MASLD was recapitulated. Male mice exhibited more severe hepatic steatosis compared to female mice, and that was markedly attenuated by intestine epithelial HIF-1α overexpression in male but not female mice, suggesting that the sex difference in MASLD is intestine epithelial HIF-1 dependent. A sex difference in HIF-1–mediated Treg response has been reported in the liver.^42^ Our data show a decreased bacterial translocation and inflammatory response in male mice, suggesting improved gut barrier function and/or altered gut microbial composition resulting from intestine epithelial HIF-1α overexpression. On the other hand, we noticed that hepatic macrophage numbers were comparable between female and male Hif1α^LSL/LSL^ mice when fed with the HFHFr diet, despite reduced hepatic fat accumulation in female mice. However, the number of F4/80-positive macrophages was not reduced in female mice by intestine HIF-1 overexpression. These seemingly paradoxical phenotypes suggest that a functional difference in hepatic macrophages may exist between male and female mice.^43^ Whether or not the subpopulations of hepatic macrophages are sex-specific warrants further investigation.

In the current study, tamoxifen was injected to induce intestine epithelial–specific HIF-1α overexpression. Recent studies suggest that tamoxifen treatment results in off-target metabolic effects independent of Cre recombinase activation–induced gene expression in a sex-dependent manner.^44^^–^^46^ Thus, tamoxifen may be a confounding factor in the sex differential effects on the improvement of MASLD by intestine epithelial HIF-1α overexpression. Although both male and female flox control mice [Cre (−)] and Cre (+) mice received an equal dose of tamoxifen, whether tamoxifen induces off-target effects in a sex-dependent manner in the context of HFHFr and intestine HIF-1α overexpression remains elusive. Given that higher basal levels of HIF-1α protein were observed in female human pulmonary artery smooth muscle cells, which increases the risk of females developing pulmonary arterial hypertension,^47^ we examined the sex difference of HIF-α activity in the intestine in HIF-α reporter mice. Interestingly, we found a higher basal ileum HIF-α activity with more pronounced alterations in response to dietary fructose and washout in female mice, likely owing to the effect of estrogen.^17^ Given the critical role of HIF in the gut barrier function, a higher basal intestine HIF activity may explain, at least partially, why females are resistant to developing MASLD. On the other hand, it is conceivable that forced intestine epithelial HIF-1α overexpression has more prominent effects on the protection against MASLD in males than in females, likely due to the sex difference in the basal intestine HIF activity. Future studies investigating sex differences in cell-specific and tissue-specific HIF activities may help to understand sex as a biological variable in health and disease.

In summary, intestine HIF-1 activation is a promising therapeutic strategy for MASLD. However, the sex differential response to intestine HIF activation in the context of MASLD must be taken into consideration.

## Supplementary Material

**Figure:**