# Supplementary material for: Intestine epithelial–specific hypoxia-inducible factor-1α overexpression ameliorates western diet–induced MASLD
Source: Hepatol Commun. 2024 Nov 25;8(12):e0572. doi: 10.1097/HC9.0000000000000572 (PMC11596589; doi:10.1097/HC9.0000000000000572)
Supplement: Supplementary file 1 [file hc9-8-e0572-s001.docx]

**SUPPLEMENTARY METHODS**

**Animal experiments**

ODD-luc mice (HIF-α luciferase reporter mice, FVB/NJ background, Jackson Lab #0062606) [^1^](#_ENREF_1), LSL-HIF1 dPA mice (Jackson Lab # 009673) [^2^](#_ENREF_2) and villin-cre-ERT2 (Jackson Lab #020282) [^3^](#_ENREF_3) were obtained from Jackson Laboratory (Bar Harbor, ME). Intestinal epithelial-specific Hif1α overexpression mice (Hif1α^LSL/LSL;VilERcre^) and wild-type littermates (Hif1α^LSL/LSL^) were generated by crossing LSL-HIF1 dPA mice with villin-cre-ERT2 mice (both are on a C57BL/6 background). The animals were group-housed in a temperature- and humidity-controlled room with a 12:12 h light-dark cycle and were on a standard pelleted rodent chow diet (5010, LabDiet). Tamoxifen was IP injected at the dose of 100mg/kg for three consecutive days to activate the Cre. Homozygous flox mice served as controls and received equal amount tamoxifen injection. After a one-week washout of tamoxifen effects, age matched (12 weeks ±) male mice were fed with high-fructose diet (HFr). Both male and female mice were fed with high-fat (D12492, Research diets, New Brunswick, NJ) and high-fructose diet (HFHFr) for 8 weeks as indicated. High fructose was given *ad libitum* via tap water containing 30% fructose (w/v) as the only water source during the entire experiment. Fructose enriched drinking water was changed twice a week. Body weight and food consumption were monitored weekly. At the end of experiment, all the animals were sacrificed under anesthesia with ketamine/xylazine (100/10mg/kg I.P. injection) after fasting for 4 hours. Blood was collected from the inferior vena cava, and citrated plasma was stored at -80°C for further analysis. Portions of liver tissue were fixed with 10% formalin, portions of distal ileum and proximal colon were fixed with Carnoy's solution for subsequent sectioning, and the other tissues were snap-frozen with liquid nitrogen.

***Ex vivo* bioluminescence imaging (BLI)**

8-10-week-old male ODD-luc mice were fed with rodent chow diet and free access to tap water or tap water containing 30% fructose or 30% glucose (w/v) as control for 2 weeks. Mice on fructose or glucose received the hydroxylase inhibitor dimethyloxalylglycine (DMOG, Cayman Chemical, Cat#: 71210) 8mg or equal volume of vehicle control via i.p. injection every second day for 2 weeks. Blood glucose was measured by glucose meter after 2 weeks treatment. At the end of experiment, the full-length GI tracts (from stomach to anus) were removed after euthanasia by cervical dislocation. Luciferase activities of entire GI tract were evaluated by bioluminescent imaging (BLI). The first image was taken in approximately 15 min post D-luciferin injection and continued every 5 mins for 45mins. D-luciferin potassium salt (Gold Biotechnology, Cat#: LUCK-1G; St Louis, MO) was IP injected at the dose of 150mg/kg BW 5mins before euthanasia. Color bar indicates photons/(ph/s/cm2/sr) with minimum and maximum threshold values [^1^](#_ENREF_1). Luciferase activity was calculated by the area under the kinetic curve (AUC) of luminescence.

**Intestine epithelium HIF-α luciferase activity assay**

8-10-weeks-old male and female ODD-luc mice were fed with rodent chow diet and free access to tap water or tap water containing 30% fructose (w/v) for two weeks. Fructose-fed mice were switched to tap water for two more weeks. At the end of experiment, all the animals were sacrificed under anesthesia with ketamine/xylazine (100/10mg/kg I.P. injection) after fasting for 4 hours. Distal ileum was harvested, and gut epithelium was scraped with slides. Luciferase activities of the ileum epithelium were measured using Promega luciferase assay system following the supplier’s instruction (Cat# E1500, Promega, Madison, WI) and normalized with protein concentration.

All animal studies were approved by the University of Louisville Institutional Animal Care and Use Committee, which is certified by the American Association of Accreditation of Laboratory Animal Care.

**Liver enzymes and plasma biochemical assay**

Liver enzymes and plasma biochemical assays were performed with com- mercially available kits: alanine aminotransferase (ALT), aspartate aminotrans-ferase (AST), triglyceride (Infinity, Thermo Electron, Melbourne, Australia); non-esterified fatty acid (NEFA) (Wako Chemicals, Richmond, VA). Plasma cytokines were measured by Proteome Profiler Mouse XL Cytokine Array following the manufacturer’s instructions (Cat# ARY028, R&D systems, Inc., Minneapolis, MN) (n=6/group, 3 different plasma samples from the same group were pulled together for the detection of cytokines in one membrane). Plasma LPS was measured by chromogenic LAL endotoxin assay kit (Cat# L00350, GenScript USA Inc., Piscataway, NJ). Plasma CCL6 (Cat# LS-F56024, LSBio, Lynnwood, WA) was measured by ELISA.

**Histology and immunohistochemistry**

Formalin-fixed, paraffin-embedded (FFPE) liver and adipose tissue sections were cut at 5 μm thickness and stained with hematoxylin and eosin (H&E). Carnoy's solution fixed, paraffin-embedded swiss-rolled distal ileum section and vertical proximal colon sections were stained with H&E and Alcian blue, respectively. Nucleus were counterstained with nuclear fast red for Alcian blue staining. For immunohistochemistry (IHC), liver and intestine sections were deparaffinized and rehydrated, followed by antigen retrieval and blocking, then incubated with primary antibody anti-F4/80 (Cat#: 70076S, Cell Signaling, Danvers, MA) for liver, anti-ki67 (Cat#: ab16667, abcam, Waltham, MA) for intestine, overnight at 4°C [^4^](#_ENREF_4). Then, the sections were incubated with horseradish peroxidase-conjugated secondary antibody for 1 h at room temperature, then stained with DAB (Cat#: SK-4105, Vector Laboratories Inc, Newark, CA, USA) for 5 min followed by counterstaining with hematoxylin, dehydration and mounting. Slides were scanned with PANNORAMIC Desk digital slide scanner (3DHISTECH Ltd., Budapest, Hungary) and images were taken with CaseViewer (3DHISTECH Ltd., Budapest, Hungary). F4/80 positive staining cells were counted on five to eight non-overlapping fields per section at 200× magnification. Crown-like structures (CLS) in adipose tissue were counted on five to ten fields per section at 100× magnification. Goblet cells were manually counted within well-oriented crypt structures in six randomly selected crypts. Villus length was measured from the tip of the villus to the base of crypt with CaseViewer [^5^](#_ENREF_5). Ki-67 positive cells were counted in 30-50 crypts per section.

**Hepatic Triglyceride Assay**

Liver tissues were homogenized in ice-cold phosphate buffered saline. Hepatic total lipids were extracted with chloroform and/or methanol (2:1) according to the method described by Bligh and Dyer [^6^](#_ENREF_6). Hepatic triglyceride content was deter- mined by commercially available kit (Infinity, Thermo Electron, Melbourne, Aus- tralia).

**Glucose tolerance test (GTT) and Insulin tolerance test (ITT)**

Mice were IP injected with 10% glucose (Sigma, St. Louis, MO, United States) at the dose of 1 g/kg body weight after 5 hours fasting at week 5 of HFr for GTT, or recombinant human insulin (Humulin R, Lilly USA, LLC, Indianapolis, IN) at the dose of 0.50 U/kg body weight after 5 hours fasting at week 7 of HFr for ITT. Glucose levels were obtained at 0, 15, 30, 60, and 120 min. Blood samples were obtained by tail nick and glucose levels were measured using an ACCU-CHEK Aviva glucose meter and test strips (Roche Diagnostics, Basel, Switzerland).

**Isolation of DNA and real time RT-PCR**

DNAs were extracted from approximately 20 mg of liver tissue using the QIAamp DNA Mini Kit (Cat# 51304, Qiagen, Germantown, MD) per the manufacturer’s instructions. Hepatic bacterial DNA was detected by quantitative PCR (qPCR) on a 7500 Real Time PCR System (Applied Biosystems, Carlsbad, CA) using universal primers for the bacterial 16S ribosomal RNA gene (Eub forward 5’-

ACTCCTACGGGAGGCAGCAG-3’, reverse 5’-ATTACCGCGGCTGCTGG-3’) and normalized to host 18S rRNA (forward 5’-CTGAGAAACGGCTACCACATC-3’, reverse 5’-GCCTCGAAAGAGTCCTGTATTG-3’) [^7^](#_ENREF_7). The relative gene expression was analyzed using the 2- ΔΔCt method.

**16S ribosomal RNA (16S rRNA) sequencing and data analysis**

Microbial genomic DNA was extracted from frozen cecum fecal samples. The composition of fecal microbiota was analyzed using Illumina MiSeq technology targeting the variable V3 and V4 regions of 16S ribosomal RNA. Quality control of raw sequence files was performed using FastQC and further analyzed using QIIME 2 (version 2019.04) as described [^8^](#_ENREF_8).

**Cecal albumin assay**

The intestinal permeability was assessed by measuring the albumin content in the cecal feces by ELISA (Cat#: ab207620, abcam, Waltham, MA) [^9^](#_ENREF_9). Feces was first diluted in 1 × PBS (10mg/ml), then further diluted in dilution buffer and analyzed following the manufacturer’s instructions.

**RNA-seq**

Ileum total RNA was extracted using RNeasy Mini Kit (Cat# 74104, Qiagen, Germantown, MD). RNA integrity was tested by Agilent 2100 bioanalyzer with RNA Integrity Number (RIN) between 7-9. Poly A enriched mRNA was used for library construction to avoid genomic DNA contamination. Constructed sequencing libraries were subject to sequencing using the Illumina Novaseq PE 150 platform (paired-end, 150 bp). Raw data (raw reads) of fastq format were firstly processed through in-house perl scripts. In this step, clean data (clean reads) were obtained by removing reads containing adapter, reads containing ploy-N and low-quality reads from raw data. Index of the reference genome was built using Hisat2 v2.0.5 and paired-end clean reads were aligned to the reference genome (ensembl_mus_musculus_grcm38_p6_gca_000001635_8) using Hisat2 v2.0.5. Feature Counts v1.5.0-p3 was used to count the reads numbers mapped to each gene, and then fragments per kilobase of transcript per million mapped reads (FPKM) of each gene was calculated based on the length of the gene and reads count mapped to this gene. Differential expression analysis of two conditions/groups (two biological replicates per condition) was performed using the DESeq2 R package (1.20.0). Genes with an adjusted P-value <0.05 found by DESeq2 were assigned as differentially expressed. Gene Ontology (GO) and Kyoto Encyclopedia of Genes and Genomes (KEGG) enrichment analysis of differentially expressed genes were implemented by the clusterProfiler R package. GO terms with corrected P value less than 0.05 were considered significantly enriched by differential expressed genes.

**Isolation of mouse intestinal crypts and growth of intestinal organoids**

Intestinal crypts were isolated and organoids were cultured using IntestiCult Organoid growth medium (Cat#06005, Stemcell Technologies, Cambridge, MA) follow supplier’s instruction as described [^10^](#_ENREF_10). Briefly, 15cm of distal small intestine, including ileum, were removed from 8-week-old male and female ODD-luc mice, opened longitudinally and washed with ice-cold PBS without Ca^++^ and Mg^++^. Then, the intestine was cut into 2 mm segments and washed extensively with cold PBS. The intestinal pieces were incubated with Gentle Cell Dissociation Reagent (Cat# 100-1077, Stemcell Technologies, Cambridge, MA) on a gently rotating platform for 15 min. Subsequently, the dissociation reagent was removed, and intestine segments were washed with PBS containing 0.1% BSA by vigorous pipetting. The washing step was repeated four times and the supernatant collected from each wash and labeled as fraction 1-4. Fractions three and four containing the intestinal crypts were pooled and filtered through a 70 μm cell strainer (Cat#542070, Greiner Bio-One, Monroe, NC). Crypts were counted under the microscope. 1,500 crypts were resuspended in 300 µL of InstestiCult Organoid growth Medium, mixed (1:1) with Corning Matrigel matrix (Cat#CLS356231, Sigma-Aldrich, St. Louis, MO), and seeded onto 24-well plates with 50µl/well, covered with 750 µl IntestiCult Organoid Growth Medium. The growth medium was changed every two or three days. On day 6, the intestinal organoids were transferred to Seahorse XFe96/XF Pro cell culture microplates (Cat#103794-100, Agilent Technologies, Inc., Santa Clara, CA) for Seahorse analysis.

**Seahorse Analysis**

5 organoids in 3 µl of Matrigel® per well were seeded onto XFe96 microplate and covered with 80 µl IntestiCult Organoid Growth Medium. After 24 hours, the organoids were treated with 1mM DMOG for an additional 24 hours [^11^](#_ENREF_11)^,^[^12^](#_ENREF_12). Then, standard glycolysis stress test was performed on Seahorse XFe96 analyzer (Agilent Technologies, Inc., Santa Clara, CA) [^13^](#_ENREF_13). Extracellular acidification rate (ECAR) was measured on addition of 10 mM glucose, 5 µm oligomycin, and 100 mM 2-DG, and normalized with protein content. The HIF-α luciferase activities of ileum organoids in response to 1mM DMOG for 24 hours were examined on 96-well cell culture plates and measured using ONE-Glo™ EX Luciferase Assay System (Cat# E8110, Promega, Madison, WI).

**REFERENCES**

1. Safran M, Kim WY, O'Connell F, et al. Mouse model for noninvasive imaging of HIF prolyl hydroxylase activity: assessment of an oral agent that stimulates erythropoietin production. *Proceedings of the National Academy of Sciences of the United States of America*. Jan 3 2006;103(1):105-10. doi:10.1073/pnas.0509459103

2. Kim WY, Safran M, Buckley MR, et al. Failure to prolyl hydroxylate hypoxia-inducible factor alpha phenocopies VHL inactivation in vivo. *The EMBO journal*. Oct 4 2006;25(19):4650-62. doi:10.1038/sj.emboj.7601300

3. el Marjou F, Janssen KP, Chang BH, et al. Tissue-specific and inducible Cre-mediated recombination in the gut epithelium. *Genesis (New York, NY : 2000)*. Jul 2004;39(3):186-93. doi:10.1002/gene.20042

4. Hou X, Yin S, Ren R, et al. Myeloid-Cell-Specific IL-6 Signaling Promotes MicroRNA-223-Enriched Exosome Production to Attenuate NAFLD-Associated Fibrosis. *Hepatology*. Jul 2021;74(1):116-132. doi:10.1002/hep.31658

5. Hasnain SZ, Wang H, Ghia JE, et al. Mucin Gene Deficiency in Mice Impairs Host Resistance to an Enteric Parasitic Infection. *Gastroenterology*. 03/17/received

01/25/accepted 2010;138(5-10):1763-1771.e5. doi:10.1053/j.gastro.2010.01.045

6. Bligh EG, Dyer WJ. A rapid method of total lipid extraction and purification. *Canadian journal of biochemistry and physiology*. Aug 1959;37(8):911-7.

7. Zhong W, Wei X, Hao L, et al. Paneth Cell Dysfunction Mediates Alcohol-related Steatohepatitis Through Promoting Bacterial Translocation in Mice: Role of Zinc Deficiency. *Hepatology*. May 2020;71(5):1575-1591. doi:10.1002/hep.30945

8. Song M, Yuan F, Li X, et al. Analysis of sex differences in dietary copper-fructose interaction-induced alterations of gut microbial activity in relation to hepatic steatosis. *Biology of sex differences*. Jan 6 2021;12(1):3. doi:10.1186/s13293-020-00346-z

9. Hartmann P, Chen P, Wang HJ, et al. Deficiency of intestinal mucin-2 ameliorates experimental alcoholic liver disease in mice. *Hepatology*. Jul 2013;58(1):108-19. doi:10.1002/hep.26321

10. Dopeso H, Rodrigues P, Cartón-García F, et al. RhoA downregulation in the murine intestinal epithelium results in chronic Wnt activation and increased tumorigenesis. *iScience*. Apr 19 2024;27(4):109400. doi:10.1016/j.isci.2024.109400

11. Okkelman IA, Neto N, Papkovsky DB, Monaghan MG, Dmitriev RI. A deeper understanding of intestinal organoid metabolism revealed by combining fluorescence lifetime imaging microscopy (FLIM) and extracellular flux analyses. *Redox biology*. Feb 2020;30:101420. doi:10.1016/j.redox.2019.101420

12. Li C, Zhou Y, Wei R, et al. Glycolytic Regulation of Intestinal Stem Cell Self-Renewal and Differentiation. *Cellular and molecular gastroenterology and hepatology*. 2023;15(4):931-947. doi:10.1016/j.jcmgh.2022.12.012

13. Ludikhuize MC, Meerlo M, Burgering BMT, Rodríguez Colman MJ. Protocol to profile the bioenergetics of organoids using Seahorse. *STAR Protoc*. Mar 19 2021;2(1):100386. doi:10.1016/j.xpro.2021.100386

**FIGURE S1** Effects of DMOG on HIF-α luciferase activity and glycolysis in female ileum organoids. (A) Representative images of ileum organoids isolated from female ODD-luc mice. Scale bar, 100 μm. (B) Female ileum organoids HIF-α luciferase activity. (C) Glycolysis stress test profile of female ileum organoids determined by Seahorse XFe96 (n=5 replicates). One representative result from 2 independent experiments is shown. (D) Glycolysis and glycolytic capacity.
